# Relationship Between Depression and Epigallocatechin Gallate from the Perspective of Gut Microbiota: A Systematic Review

**DOI:** 10.3390/nu17020259

**Published:** 2025-01-12

**Authors:** Yangbo Zhang, Changwei Liu, Qi Zhu, Hui Wu, Zhonghua Liu, Li Zeng

**Affiliations:** 1 School of Pharmacy, Shaoyang University, Shaoyang 422000, China; zhangyblucky@163.com (Y.Z.); qier27@yeah.net (Q.Z.); 19156233156@163.com (H.W.); 2 Key Laboratory of Tea Science of Ministry of Education, Hunan Agricultural University, Changsha 410128, China; lcvv666@163.com; 3 School of Life and Health Sciences, Hunan University of Science and Technology, Xiangtan 411201, China; 4 National Research Center of Engineering and Technology for Utilization of Botanical Functional Ingredients, Hunan Agricultural University, Changsha 410128, China; 5 Co-Innovation Center of Education Ministry for Utilization of Botanical Functional Ingredients, Hunan Agricultural University, Changsha 410128, China

**Keywords:** depression, gut microbes, gut–brain axis, EGCG

## Abstract

Depression, a serious mental illness, is characterized by high risk, high incidence, persistence, and tendency to relapse, posing a significant burden on global health. The connection between depression and gut microbiota is an emerging field of study in psychiatry and neuroscience. Understanding the gut–brain axis is pivotal for understanding the pathogenesis and treatment of depression. Gut microbes influence depression-like behaviors by impacting the hypothalamic–pituitary–adrenal axis (HPA), monoamine neurotransmitters, immune responses, cell signaling, and metabolic pathways. Tea, widely used in clinical practice to improve neuropsychiatric disorders, contains Epigallocatechin gallate (EGCG), a major ingredient of green tea, which effectively regulates intestinal flora. This review examined the risks and causes of depression, the complications associated with intestinal flora, their role in the development and treatment of depression, and how EGCG may alleviate depression through interactions with gut microbiota and other mechanisms.

## 1. Introduction

Depression is a prevalent and serious mental illness that imposes a prominent global health burden. According to the World Health Organization (WHO), over 350 million people worldwide suffer from depression, making it one of the most concerning disorders due to its high rates of disability, morbidity, and mortality [1,2,3,4]. Symptoms of depression include reduced interest or pleasure in activities, slowed thinking, low mood, decreased activity, and impaired concentration. The disorder is characterized by its high risk, incidence, persistence, and tendency for recurrence.

**Figure 1:**
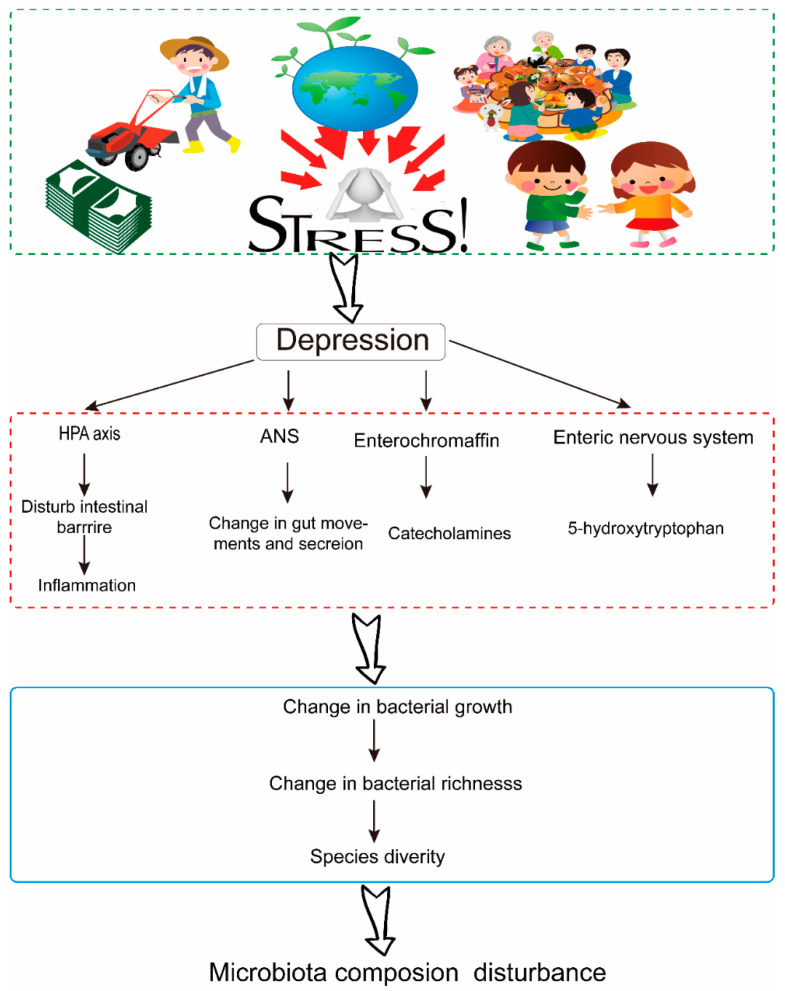
Mechanisms of depression’s effect on gut microbiota. The dash-line box in green indicates the kind of social causes of depression. The dash-line box in red indicates the relationship between biochemical indicators of depression and gut microbiota. The dash-line box in blue indicates the changes the gut microbiota.

With increasing stress and unhealthy lifestyle choices, the prevalence of major depressive disorder (MDD) is rising rapidly. As the number of individuals affected by depression continues to grow, there is an urgent necessity to comprehend the underlying mechanisms of the disease in order to develop effective treatments. The etiology of depression is multifaceted, involving genetic, psychological, and social factors, disruptions in biological rhythms, physical illnesses, and substance abuse. Previous research has identified several pathogenic mechanisms: (1) the monoamine hypothesis suggests a deficiency in biogenic amine systems, particularly serotonin (5-HT) and norepinephrine (NE); (2) the hypothalamic–pituitary–adrenal (HPA) axis dysfunction theory related to hyperactivation of the HPA, resulting in elevated levels of neuroendocrine hormones; (3) the neurogenesis and neurodegeneration hypothesis involves decreased neurogenesis and the onset of neurodegeneration; (4) the inflammatory theory suggests dysfunction in peripheral immune responses and implicates neuro-immunological mechanisms [5,6,7,8,9,10]. In summary, the primary mechanisms implicated in the onset of depression include HPA hyperactivity, inflammation, neurogenesis/neuroplasticity, the monoamine system, and the gut–brain axis (GBA) (Figure 1) [11,12,13].

However, the development of treatments for MDD and the improvement of their efficacy have been limited. Antidepressants and anxiolytics, while effective, often come with undesirable side effects [12,14]. There is a pressing need to identify more effective therapeutic approaches to alleviate or prevent depression. Growing research indicates that adopting a healthy lifestyle can significantly reduce the risk of MDD. Specifically, behaviors such as reducing smoking [15], moderating alcohol consumption [16], maintaining a low-fat diet [17], and engaging in regular physical exercise [18], have demonstrated beneficial effects. Moreover, several studies have highlighted a strong correlation between regular tea consumption and a lower prevalence of psychological disorders, such as depression [19,20,21]. This association may be attributed to bioactive compounds found in tea, such as tea polyphenols [22], epigallocatechin gallate (EGCG) [23], and L-theanine [24]. This review aimed to elucidate the influences of intestinal flora in the mechanisms and treatment of depression, as well as to explore the underlying contributions of tea and its bioactive ingredients, particularly EGCG, in alleviating and managing depressive symptoms.

## 2. The Relationship Between Microbiome and Depression

Recently published research manifests that the composition and diversity of gut bacteria can significantly impact psychological health, including depression, anxiety, etc. Changes in intestinal flora composition can influence brain function and behavior [14], including emotion regulation, stress response, and potentially the onset of psychiatric disorders like depression [15]. The GBA is defined as the bidirectional communication network between the gut and the brain. The intestinal flora, which comprises the microbial community that lives in the digestive tract, plays a pivotal role in this axis. The concept of the GBA underscores the bidirectional regulatory relationship between the brain and gut microbes in the development of depression, providing new perspectives on its treatment [16,17]. Therefore, maintaining or restoring a healthy balance of intestinal flora is vital for preventing and treating psychological disorders [18].

The gut microbiota plays a critical role in human health and disease, serving as a key regulator of host physiology [19]. Dysregulation of intestinal flora has been implicated in the mechanisms and treatment of chronic pain and depression [20]. Changes in gut microbiota are related to a number of mental and neurodegenerative disorders, like schizophrenia, depression, and Alzheimer’s disease [21,22,23]. The diversity of the intestinal flora is mightily associated with behaviors related to emotions, such as MDD, characterized by a bidirectional connection between the gut and brain regulated by pathways such as neuroendocrine, neuroimmune, and sensory neural pathways [17]. Gut microbiota not only contribute to the causes of depression but also influence the mechanisms of antidepressants in vivo through interactions with intestinal flora and enterohepatic circulation. This interaction may potentially reduce drug resistance in depressed patients.

Depression is in connection with changes in the composition of gut microorganisms [24]. Pressure has been shown to cause an imbalance of human gut microbiota, leading to mental health problems like anxiety and depression [25,26]. The onsets of depression-like moods have been shown to change the structure and number of intestinal flora. Additionally, anxiety and depression are often accompanied by altering colonic motility, resulting in the composition and stability of gut microbiota being disrupted and changes in gut ecology and morphology. Research has indicated that chronic unpredictable mild stress (CUMS) treatment altered the diversity and composition of intestinal flora and increased the intestinal epithelium’s permeability, suggesting a defect happened in the intestinal barrier [17]. The disruption of mucosal barrier integrity induced by CUMS is linked to reduce the concentration of tight junction protein occludin-1 and impaired mucosal layer function due to reductions in goblet cell activity. The onset of depression and anxiety has a significant impact on the microbial community’s composition within the colon. It indicates such changes are triggered by heightened activation of the stress response, alongside disruptions to the microbial environment resulting from variations in colonic motility [27].

## 3. The Composition of the Gut Microbiome in Depression

It is estimated that the intestinal flora consists of trillions of microbes, encompassing over 100 species of bacteria. These microorganisms are primarily categorized into six phyla: *Firmicutes* (like *Enterococcus*, *Clostridium*, *Ruminococcus*, and *Lactobacillus*, making up 60%), *Bacteroidetes* (including *Prevotella*, and *Bacteroides* 15%), *Actinobacteria*, *Proteobacteria*, *Fusobacteria*, and *Verrucomicrobia* [28]. In mammals, altering the gut microbiota’s composition is implicated in various physiological processes, including development, immune cell function, and metabolism, and is connected with susceptibility to numerous diseases [29]. The composition and diversity of gut microbiota have been linked to a broad range of diseases, encompassing gastrointestinal issues, for example, *Clostridium* difficile infection, as well as various other health conditions such as depression, anxiety, cardiovascular disease, type 2 diabetes mellitus, and rheumatoid arthritis [30]. The host and its microbial inhabitants can be defined as a “superorganism” performing immune and metabolic functions due to the stability, diversity, and resilience of gut microbes, which interact symbiotically with its host. These symbiotic microbiotas also interact closely with the immune system in the intestinal mucosa, helping maintain mucosal barrier integrity and intestinal epithelial function, thereby preventing bacterial infiltration. The gut microbiome significantly influences the central nervous system’s (CNS) development, consisting of a vast community of bacteria and viruses that significantly impact the physical condition of the host [31]. Dysregulation of the gut flora can contribute to neurodegenerative diseases. The gut microbiota is connected with the brain through a number of metabolites produced by specific gut cells, including intestinal endocrine cells (ECCs) and afferent nerve endings [32].

The gut flora has been shown to modulate behaviors associated with mental illness, like anxiety and depressive behaviors [12]. Under normal circumstances, the host maintains delicate homeostasis with its gut microbiome through immune regulation, nutrient supply, and respiratory chain regulation. However, stress can disrupt this homeostasis, leading to changes in the structure and function of the gut microbiome. In individuals with post-stroke depression (PSD), there is a decrease in species richness indices, characterized by the depletion of 22 Operational Taxonomic Units (OTUs), mainly from the phylum *Firmicutes*, including genera like *Blautia* and *Streptococcus* [33]. The production of 5-HT in the colon, which is crucial for mood regulation, is significantly influenced by gut microbiota, especially metabolites from spore-forming bacteria [34]. In rodent models induced by CUMS over three weeks, a significant shift in beta-diversity occurs, with an increased *Firmicutes* to *Bacteroidetes* (F/B) ratio because the abundance of *Bacteroidetes* was decreased, and the abundance of *Firmicutes* was elevated [4,35]. Similar changes in microbial composition were observed in CUMS-treated mice, with a significant decrease in *Bacteroidetes* and an increase in *Firmicutes* at the phylum level [36], particularly affecting genera like *Porphyromonadaceae* and *Barnesiella*, known for their protective properties [35]. Studies comparing the gut microbiota of MDD patients to healthy ones consistently showed prominent differences in the relative abundance of *Firmicutes*, *Actinobacteria*, and *Bacteroidetes* [21]. At the genus level, interventions like mild stress have been shown to increase the abundance of *Odoribacter* while decreasing *Lactobacillus* and the *Lachnospiraceae_NK4A136* group [24]. Comparatively, individuals with depression exhibit an increased abundance of *Firmicutes*, *Proteobacteria*, and *Actinobacteria*, while showing a reduction in *Epsilonbacteraeota* and *Bacteroidetes* [24]. The gut microbiota of CUMS donor and recipient models differs from the control, which is characterized by a higher relative abundance of *Akkermansia* and a lower relative abundance of *Lactobacillus* in CUMS group [14]. Depressed patients typically show higher levels of *Proteobacteria* abundance and lower levels of *Clostridium sphaeroides* and *Bacteroides fragilis* [37,38]. At the phylum level, *Actinobacteria* and *Proteobacteria* are elevated in fecal samples from MDD patients compared to healthy ones, while *Bacteroidetes* and *Firmicutes* are reduced. Additionally, the abundance of *Actinomycetes* has a positive relationship with the seriousness of MDD. Compared to healthy controls, depressed patients exhibit decreased levels of *Clostridium*, *Erysipelotrichaceae*, and *Turicibacter*, and increased levels of *Veillonellaceae* in their gut microbiota [39].

Zhang Jinna [40] observed significant differences in the intestinal flora structure between FH/Wjd depressive rats and SD rats. Depressed rats showed decreased levels of *Ruminococcus* and thirteen other genera of bacteria, while five genera, including *Rikenella*, *Oscillibacter*, and *Anaeroplasma* were increased. As the FH/Wjd depressive rats aged, further changes in their gut microbiota occurred, including increases in *Acinetobacter*, *Oscillibacter*, *Bacillus*, and *Bilophila.* The occurrence of depression in this context may be connected with reductions in beneficial gut bacteria and increases in harmful ones. Jiang Haiyin [41] found that depressive patients exhibited elevated proportions of *Bacteroidetes*, *Proteobacteria*, and *Actinobacteria* at the phylum level, with a decrease in *Firmicutes*. At the family level, *Acidaminococaceae*, *Enterobacteriaceae*, *Fusobacteriaceae*, *Porphyromonadaceae*, and *Rikenellaceae* had a higher abundant in the gut of depressed patients; however, *Bacteroidaceae*, *Erysipelotrichaceae*, *Lachnospiraceae*, *Prevotellaceae*, *Ruminococcaceae*, and *Veillonellaceae* were reduced. Particularly, *Lachnospiraceae* and *Ruminococcaceae* were significantly diminished in depressed patients. The changes in rat behaviors caused by stress may be related to species within *Lachnospiraceae* and *Ruminococcaceae*. The lower abundance of *Lachnospiraceae* in the gut may be related to an increase in the intestinal mucosal permeability observed in depressed patients. In the depressive model of rats following myocardial infarction, probiotics like *Lactobacillus* and *Bifidobacterium* exhibited antidepressant effects by reducing inflammatory markers and restoring intestinal barrier integrity [15,42,43,44].

## 4. Molecular Mechanism of Intestinal Microbiome Disturbance Inducing Depression via the Microbial-GBA

**Figure 2:**
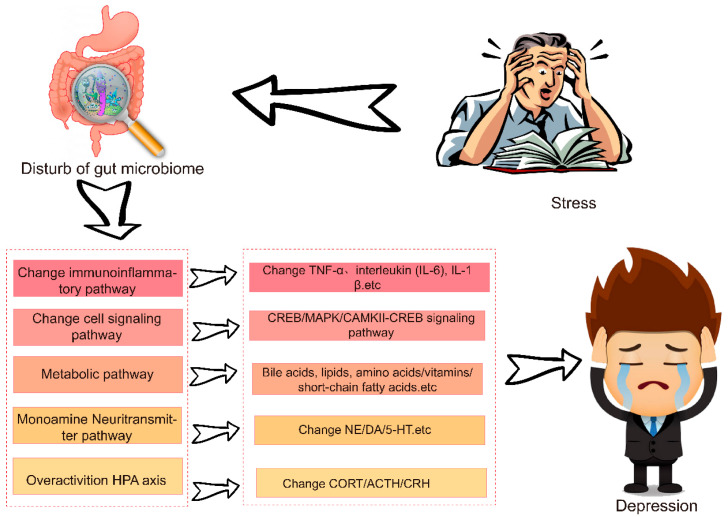
Molecular mechanism of intestinal microbiome disturbance inducing depression.

The gastrointestinal microbiota has a dynamic effect in maintaining various physiological functions in the human body, including preserving the gastrointestinal epithelial barrier, defending against pathogens, promoting nutrient absorption, and regulating digestive, metabolic, and neuroimmune processes [11]. Gut microbes are crucial for brain development, function, behavior, host metabolism, infection resistance, and immune system maturation. Disruptions or absence of intestinal microbiota can negatively affect neurogenesis, cortical myelination, blood–brain barrier (BBB) integrity, microglial maturation, social behaviors, stress responses, and fear learning [12,29]. Changes in brain vascular physiology, brain structure, and BBB permeability are critical factors contributing to neurological dysfunctions [45], highlighting the influence of disrupted gut microbiota on depression. How does the dysregulation of gut microbes induce depression? Gut microorganisms influence the brain through at least five pathways: the HPA, monoamine neurotransmitter pathways, immunoinflammatory pathways, cell signaling, and microbial metabolites (Figure 2). The GBA influences nerve and glial cells, neurotransmitters, brain-derived neurotrophic factors, inflammatory factors, short-chain fatty acids (SCFAs), circulating metabolites, the BBB, and oxidative stress [46]. Disruptions in GBA functions can lead to dysfunctions in the brain, immune system, endocrine system, and gut, which in turn can affect the neuropsychological functions of the CNS [24]. There are several potential pathways through which the gut microbiota communicates with the brain, including complex innervation systems and small-molecule signaling via the GBA. This axis is implicated in maintaining homeostasis and contributes to the pathogenesis of various diseases, including neurological, degenerative, and autoimmune conditions. Activation of the GBA involves the immune response, neurotransmitter signaling, and hormonal systems [47]. It is believed that the microbiota-GBA regulates various central processes Via mechanisms such as the production of microbial metabolites, immune mediators, and the vagus nerve that influence neurotransmission, neuroinflammation, and behavior.

### 4.1. The Gut Microbiome Altered Depression by HPA

**Figure 3:**
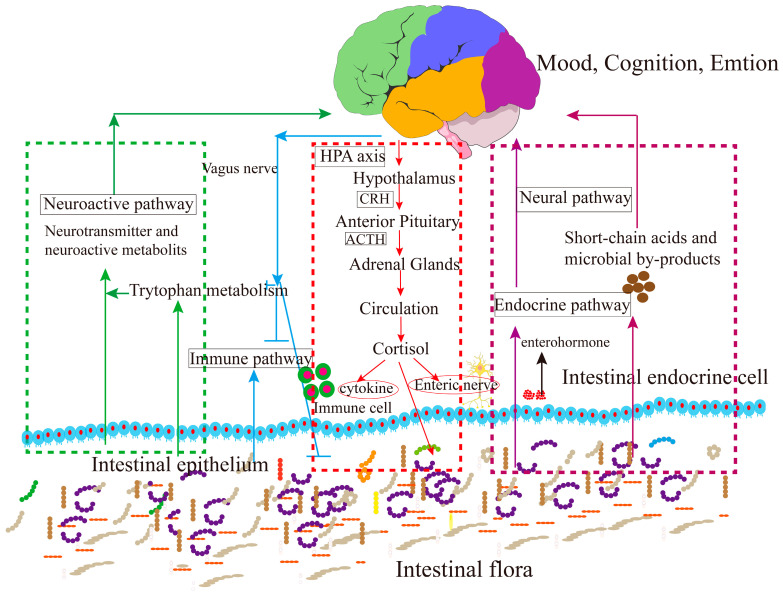
The interaction between gut flora and the brain. Arrow in green indicates the neuroactive pathway; arrow in blue indicates the immune pathway; arrow in red indicates the HPA axis; arrow in purple indicates the metabolic pathway.

The HPA is a crucial component of the neuroendocrine system and plays a crucial role in the pathogenesis of mood disorders. Stress signals activate pathways through the sympathetic nervous system (SNS) and/or the HPA. SNS activation leads to the release of hormones from the adrenal medulla and NE from nerve endings, affecting various organs. The dysregulation of the HPA is usually observed in individuals with depression. Gut microbiota impact the HPA by regulating the production and stress hormones, like cortisol, which in turn impact mood and behavior (Figure 3). In individuals with depression or chronic stress, the HPA may become dysregulated, resulting in excessive cortisol circulation. Dysfunction of the HPA, characterized by increased cortisol, adrenocorticotropic hormone (ACTH), and corticotropin-releasing hormone (CRH), is pivotal in the pathogenesis of MDD [48,49]. This overactivation releases glucocorticoids, which typically have immunosuppressive effects. Elevated glucocorticoid levels under stress conditions can cause apoptosis in B and T lymphocytes, impairing adaptive immunity and potentially leading to depression. Compared to specific pathogen-free (SPF) mice, the expression of genes related to the glucocorticoid receptor pathway (e.g., *Aqp1*, *Slc22a5*, *Ampd3*, *Stat5a*, *Cyb561*, *Plekhf1*) was up-regulated in the hippocampus of germ-free (GF) mice, suggesting that the absence of gut microbes may induce depressive and anxious behaviors by disrupting the function of glucocorticoid receptors [50].

Signaling from the brain via the HPA also affects various gastrointestinal processes, including motility, fluid and mucus secretion, intestinal permeability, immune activation, and the relative abundance, and expression patterns in specific gut microbiota [32]. These findings suggest that gut microbes influence depression through the HPA. Chronic depression can alter motor activity and microbial distribution in the colon via HPA activation. Emerging evidence suggests that dynamic changes in intestinal flora can affect brain physiology and behavior. For instance, acute stress in GF mice led to significantly elevated plasma corticosterone (CORT) and ACTH levels [51,52], but these levels normalized after transplanting normal fecal microbiota into the GF mice, highlighting the crucial role of gut microbiota in regulating HPA activity during stress [53]. Disturbances in gut microbiota may induce depressive and anxiety-like behaviors, and changes in gene expression associated with the HPA. In mice with depression-like phenotypes, changes in the protein expression levels in multiple tissues of the GBA were observed, and these changes were specific to the chronic stress model [54]. The hippocampus, crucial for memory and learning, plays a role in modulating the HPA and stress responses. Gut microbes are related to the volume of the hippocampus and amygdala, regions involved in mood, appetite, and fear. Slow development of the hippocampus may impair stress responses. Certain gut microbes, such as *Lactobacillus* and *Bifidobacterium*, regulate cortisol levels, potentially reducing negative emotions, improving cognition, alleviating irritable bowel syndrome symptoms, and even treating depression. The intestinal flora is also involved in the development of the HPA, regulating stress responses and cortisol release.

### 4.2. The Gut Microbiome Altered Depression by Monoamine Neurotransmitter Pathway

The intestinal flora plays a key role in the synthesis of neurotransmitters like dopamine (DA), 5-HT, and gamma-aminobutyric acid (GABA), all of which are critical for mood regulation and can influence the development of depression. The GBA acts as a biochemical link that connects the CNS and the enteric nervous system (ENS) [55], both of which are essential for maintaining human homeostasis, including immunity, nutrition, and metabolism. The interaction between the ENS, CNS, and gut microbiota regulates nutrient metabolism through ECCs that produce hormones like peptide YY (PYY), cholecystokinin (CCK), leptin, ghrelin, 5-HT, and glucagon-like peptide-1 (GLP-1) [28]. Depletion of monoamine neurotransmitters is an important risk factor for depression. Current antidepressant medications primarily target the biogenic amine hypothesis, aiming to increase the concentrations of neurotransmitters like DA, 5-HT, and NE in synaptic gaps by inhibiting relevant receptors or enzymes [56]. In GF mice, compared to SPF mice, expression levels of 5-HT and DA receptors (*Htr3b*, *Htr1f*, *Drd3*, and *Htr7*), monoamine neurotransmitter transporters, metabolic enzymes (*Tdo2*, *Maob*, and *Ddc*), and related signaling molecules (*Creb1*, *Fos*, *Akt1*, *Pik3ca*, *Gsk3a*, *Pla2g5*, *Grk6*, and *Cyp2d22*) were significantly elevated [57]. Expression levels of downstream signaling molecules in 5-HT and DA synapses (*Ephb1*, *Slc18a1*, *Nr4a1*, and *Gdnf*) were also significantly changed in GF mice, returning to normal levels after recolonization with intestinal microbes. This suggests that the gut microbiome may affect brain function and behavior by regulating the monoamine neurotransmitter system. Additionally, gut microbes can affect tryptophan metabolism through the kynurenine pathway, reducing 5-HT production Via the activating indoleamine-2,3-dioxygenase (IDO). Reduced 5-HT production has been associated with depressive-like behavior, whereas increasing 5-HT levels can ameliorate such behavior [58].

Neuropeptides also play a vital role in maintaining intestinal homeostasis and influencing the composition of intestinal flora. Changes in neuropeptide levels have been implicated in various intestinal disorders and neuropsychiatric conditions associated with gut inflammation. Disruptions in gut microbiome diversity can contribute to increased intestinal permeability and systemic inflammation, both of which are connected to the CNS [59]. Gut microbes affect brain function and behavior mainly through the release of chemicals that can cross the BBB, influencing the growth, function, and connectivity of neurons. Some of these chemicals also interact with immune cells in the brain. The ENS, composed primarily of enteric glial cells that resemble astrocytes in the CNS, synthesizes neuropeptides within the intestine and signals to distant organs, such as the brain, through receptors on the vagus nerve. The intestinal flora can interact with the vagus nerve, influencing mood, stress responses, and other brain functions. Dysregulation of this communication pathway may lead to depression. Intestinal epithelial cells, located in the intestinal wall and mucous membrane lamina propria, autonomously regulate the physiology and function of the gastrointestinal tract. Through bidirectional communication via the vagus nerve, this system forms the GBA, promoting the connection between the gut and CNS.

The gut microbiota interacts with CNS through various pathways, including vagal, endocrine, and immune pathways [60]. It plays a crucial role in the development and function of microglia and astrocytes, neural-development, neurotransmitter regulation, immune activation in the CNS, and physiological processes such as maintaining BBB integrity [19]. Certain epithelial cells lining the intestinal wall have endocrine or paracrine functions that can affect the production of neurotransmitters or their precursors. The gut microbiota can produce several neurotransmitters that regulate mood, anxiety, and cognitive functions. For example, *Bifidobacterium* and *Lactobacillus* synthesize GABA, while *Escherichia* produces NE, 5-HT, and DA. Species like *Streptococcus* and *Enterococcus* are involved in 5-HT synthesis, and *Bacillus* synthesizes NE and DA [12]. The gut microbiota significantly impacts processes connected with neurotransmitter formation, neuronal myelination in the prefrontal cortex (PFC), and the development of brain regions like the amygdala and hippocampus. Changes in the relative abundance of intestinal bacteria have been related to altered levels of the brain-derived neurotrophic factor (BDNF) in the hippocampus and behavioral changes [27]. The gut microbiota also regulates the serotonergic system, affecting levels of 5-HT and related metabolites in the limbic system of GF animals. Additionally, gut flora can influence the activity of various oxidoreductases involved in neurotransmitter metabolism. For example, laccase enzymes regulate 5-HT levels in the gut and are involved in its metabolism, playing a crucial role in the GBA. Laccase enzymes oxidize catecholamines into reactive oxygen species and 3,4-dihydroxyphenylacetaldehyde quinone (DAQ), processes are associated with mitochondrial function impairment and dementia in Parkinson’s disease. Lipopolysaccharide (LPS) signaling via toll-like receptors mediate communication between the gut flora and the ENS. Psychological illnesses, such as autism and major depression, have been linked to altered circulating levels of neurotransmitters like neuropeptide Y, substance P, and calcitonin gene-related peptides.

### 4.3. The Gut Microbiome Altered Depression by Immunoinflammatory Pathway

Chronic inflammation has been associated with the formation of depression and other mood disorders. The dysregulation of inflammatory responses is referred to as the mechanism of MDD, with elevated inflammatory markers increasing susceptibility to MDD [61]. Stress-related psychiatric disorders can change the integrity of the gut barrier, potentially weakening the gut. It can result from the transfer of certain microbes or the production of microbiota-driven pro-inflammatory responses. In MDD patients, concentrations of pro-inflammatory cytokines, such as interleukin (IL)-1β and tumor necrosis factor-alpha (TNF-α), are significantly increased, while anti-inflammatory cytokines, like IL-4 and IL-6, are decreased [62]. Depression disrupts the GBA, influencing gut functions like secretion and motility, leading to visceral allergies and alterations in the intestinal endocrine and immune systems.

Gut microbiota is crucial for maintaining gastrointestinal function and influencing brain activity and behavior through immune modulation [63,64]. Imbalances in gut microbiota can lead to heightened inflammation and immune system activation. Microbiota-derived signals influence immune cells, which, in turn, impact brain function and behavior [29]. The integrity of the gut barrier is essential for preserving intestinal health. When the barrier is compromised, often referred to as “leaky gut”, harmful substances can enter the bloodstream, triggering an inflammatory cascade. Disruption of gut microbial composition may increase intestinal permeability, activate systemic immune responses, and damage the BBB, contributing to neuroinflammation and injury. Microbial metabolites and molecules released by gut microbes can stimulate host-derived cytokines and inflammation in the CNS, contributing to brain disorders such as depression, pain, autism, anxiety, Parkinson’s disease, Alzheimer’s disease, and stroke. Disturbances in gut microbiota can lead to abnormal levels of inflammatory factors and immune regulators in the development of CNS, potentially predisposing individuals to depression-like symptoms. Moreover, gut microbes may connect the ENS and CNS through the GBA. Disruption of the structure of intestinal microbial flora may cause depression-like mood changes through the GBA, potentially affecting inflammatory mediators, neurotransmitter release, and synaptic plasticity. Gut microbiota can regulate inflammation induced by psychological pressure. External stimuli that alter gut microbes may aggrandize trigger systemic inflammation, immune responses, and the permeability, influence the synthesis and efficacy of monoamine neurotransmitters, affect the activity and function of the HPA, alter BDNF levels, and ultimately contribute to the development of depression [65].

Immune cells actively monitor symbiotic bacteria within the intestine, influencing functions both locally and distantly. For example, probiotics like *Lactobacillus* and *Bifidobacterium* demonstrate antidepressant effects by decreasing inflammatory markers and restoring the integrity of intestinal barrier in a rat model of depression following myocardial infarction. Analysis of intestinal microorganisms in MDD reveals decreased abundance of anti-inflammatory microbes such as *Coprococcus* and *Faecalibacterium*, while pro-inflammatory microbes like *Eggerthella* are increased [66]. The recognition of bacterial metabolites by immune cells triggers neural signaling to the brain and interacts with intestinal immune cells, resulting in localized and systemic immune responses. These metabolites can also reach concentrations high enough to influence brain circuits by traversing the BBB. The intestinal glial network, which regulates gastrointestinal functions (such as blood flow, intestinal motility, immune responses, and exocrine/endocrine activities), relies on calcium-dependent signaling facilitated by intestinal flora. Disruptions in intestinal glial cells can lead to gastrointestinal disorders such as inflammatory bowel disease, movement disorders, neurodegenerative conditions like Parkinson’s disease, and intestinal inflammation due to infections. *Bacteroides thetaiotaomicron* exerts protective effects through its anti-inflammatory activity via NF-κB signaling in epithelial cells. Additionally, an increased abundance of *Akkermansia muciniphila* and decreased *Prevotella* levels are associated with enhanced mucin degradation, potentially contributing to increased intestinal permeability observed in patients with Parkinson’s disease.

### 4.4. The Gut Microbiome Altered Depression by Cell Signaling Pathway

Disruptions in cell signaling pathways are closely connected with the pathogenesis of MDD. One such pathway, the cAMP response element-binding (CREB) signaling pathway, also has a closer relationship with microbiome-induced depression [67]. Comparative analysis of transcriptomic changes (including long non-coding RNAs (lncRNAs), microRNAs (miRNAs), and messenger RNAs (mRNAs)) in hippocampal tissues from GF and SPF mice before and after gut microbiome recolonization revealed that the gut microbiome can regulate brain function and behavior through the CREB and Ras/mitogen-activated protein kinases (MAPK) signaling pathways [68]. A proteomic analysis of acetylated and succinylated protein modifications in the hippocampal tissues of mice undergoing intestinal flora transplantation demonstrated that intestinal microbial imbalances could regulate the activity of the MAPK signaling pathway by changing the protein levels related to post-translational modifications, thereby inducing depression-like behavior in mice [69]. The calcium/calmodulin-dependent protein kinase II (CAMK II)-CREB signaling pathway played a vital role in mediating the effects of gut microbiota on brain function. Subsequent studies using intestinal flora transplantation confirmed that this pathway is a critical component of molecular mechanisms linking the gut–brain axis to microbiome-induced depression [70]. Intestinal flora interacts with intestinal cells, the ENS, and the CNS, through metabolic and neuroendocrine pathways. In a mouse model, a low-folate diet resulted in motor symptoms resembling Parkinson’s disease, along with increased homocysteine levels, suggesting that folic acid indirectly affects dopaminergic neurons.

### 4.5. The Gut Microbiome Altered Depression by Metabolic Pathway

Gut microbes perform essential functions in the body, including protecting the host from pathogenic microorganisms, providing essential nutrients, facilitating the metabolism of certain drugs, and aiding in the absorption and storage of fatty acids [71]. By maintaining a dynamic balance, the gut microbiome plays a vital role in human health by maintaining nutrient absorption, immune function, and host metabolism [24]. By influencing nutrient absorption and metabolism, gut microbes can impact brain function. Host-dependent microbes may make the host more susceptible to environmental changes [72]. Gut bacteria are pivotal for gastrointestinal digestion, playing key roles in extracting, synthesizing, and absorbing various nutrients and metabolites such as lipids, bile acids, vitamins, amino acids, and SCFAs. These microbial metabolites, produced when bacteria metabolize food, can influence brain function. SCFAs like butyric acid, a primary gut microbiome metabolite, can reach the brain, and influence its growth and function. Specific gut flora is essential for the production and regulation of neurotransmitters and metabolites, including 5-HT, DA, and GABA. LPS produced by gut bacteria contributes to inflammation in individuals with depression [12]. Jian Yang et al. identified differences between MDD patients and healthy individuals, including variations in bacteriophages, bacterial species, and fecal metabolites [31]. MDD patients showed an increased abundance of *Bacteroides* in the genus and a decreased abundance of *Eubacterium* and *Blautia*. Disruptions in microbial genes and fecal metabolites were consistently related to amino acid metabolism, including phenylalanine, γ-aminobutyrate, and tryptophan [31].

Certain chemicals synthesized in the gut, such as somatostatin and CCK, are also produced by brain cells, and play a significant role in maintaining brain function. Evidence strongly links gut microbiota composition with host metabolism [73]. Disturbances in the intestinal microbiome can induce depression by causing dysfunction in microbial genetics and the metabolism [21]. Gut bacteria produce SCFAs via the dietary fiber fermentation, with metabolites like tryptophan-derived metabolites, and secondary bile acids also playing crucial roles in depression [20]. SCFAs possess anti-inflammatory and neuroprotective effects and may modulate brain function and mood by affecting the ENS, stimulating the SNS, releasing mucosal 5-HT, and influencing memory and learning. SCFAs also affect the CNS via immune, endocrine, and vagal pathways [32]. These SCFAs are critical for intestinal health; they are derived from the fermentation or degradation of carbohydrates and proteins in dietary fiber by gut microbiota, and have emerged as key mediators of the gut–brain-microbiota axis signaling [74]. SCFAs are not only a vital energy source for intestinal epithelial cells, but they also affect the intestinal mucosal barrier, epithelial cell permeability, and oxidative stress responses, thereby playing an important role in colon health. Genera such as *Roseburia*, *Blautia*, and *Lachnospiracease incertae sedis* contribute to the breakdown of carbohydrates into SCFAs, and reductions in these bacteria typically lower SCFA levels. Increased SCFA production can stimulate ECCs to release GLP-1 and PYY while decreasing ghrelin secretion [32]. Furthermore, SCFAs also promote the secretion of 5-HT, enhance colonic contractions, and accelerate colonic transit. SCFAs produced through intestinal microbial metabolism could regulate the level of 5-HT in vivo, with decreases in 5-HT levels directly contributing to MDD.

Intestinal microbial dysfunctions can influence brain function by affecting energy metabolism and the tryptophan metabolism pathway in the liver [75]. Serum metabolomics studies in depressed rats have revealed alterations in amino acid, glucose, and lipid metabolism. Analysis of hippocampal metabolites in these animals showed disruptions primarily in carbon and amino acid metabolism [75]. Furthermore, disruptions in the intestinal microbiota have been associated with significant changes in protein phosphorylation modifications in the hippocampus, suggesting a potential mechanism by which microbial disorders induce depression [37]. Data integration from phosphorylated proteins and metabolites in various models, including “humanized” mouse depression models and postmortem brain tissues from depressed patients, suggested that disorders in lipid and amino acid metabolism, along with axon-oriented function, may mediate intestinal microbiome-induced depression. In patients with MDD and post-traumatic stress disorder, impaired olfactory function has been related to a reduced olfactory bulb and decreased gene expression in olfactory pathways. This impairment may be related to disruptions in glycolysis, amino acid metabolism, the tricarboxylic acid cycle, and purine metabolism. Research suggests bidirectional communication between the brain and gut, where stress signals from the insula to the gut can trigger responses like stomach ulcers under chronic stress. Alterations in gut microbiota due to chronic stress can lower lipid metabolites in the blood and brain, particularly endocannabinoids, which are crucial for coordinating signal regulation in the body. *Endocannabinoids* bind to specific receptors in the hippocampus and play an important role in the formation of memories and emotions. Chronic stress-induced reductions in endocannabinoid levels may impair this signaling system, contributing to depressive behaviors [76].

The relationship between gut microbiota imbalance and depression primarily involves three key aspects: Firstly, depression is related to tryptophan metabolism. Micro-organisms may affect this process by activating IDO, an enzyme that depletes tryptophan through the kynurenine pathway, leading to decreasing 5-HT levels and triggering depressive symptoms. Tryptophan is a precursor to 5-HT, an important neurotransmitter associated with various behavioral issues. Additionally, the kynurenine pathway produces neurotoxic metabolites like quinolinic acid, which can damage nerves. Secondly, intestinal flora impacts the host’s nutrient absorption and metabolism, particularly in the digestion and absorption of carbohydrates. Thirdly, stressful events, both psychosocial and physical stressors, can trigger depression by altering gut microbiota composition, leading to reduced microbial diversity, including a decline in beneficial species like *Lactobacillus* and *Bifidobacterium* [42,77,78]. The contribution of gut microbiota to depression primarily involves microbially derived molecules, such as neurotransmitters, bile acids, indoles, SCFAs, lactate, choline metabolites, and vitamins—that influence mental behaviors.

## 5. The Relationship Between Tea and Depression

**Figure 4:**
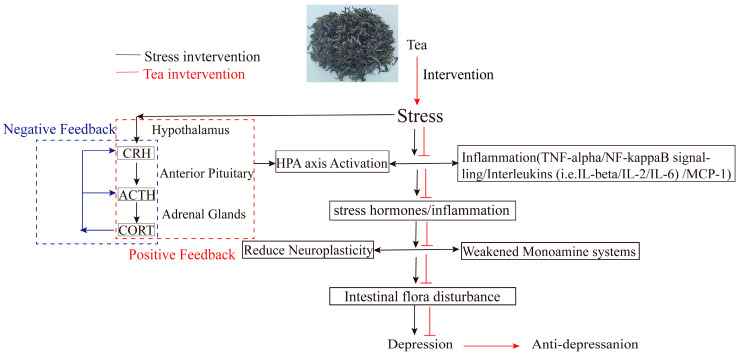
The mechanism of regulating-depression behavior after the intervention of tea.

Tea, containing bioactive ingredients, like tea polyphenols, L-theanine, flavonoids, etc., exerts positive influences on inflammation, metabolism, and the endocrine system, potentially reducing the risk of depression. Mechanistic studies, including animal models, epidemiological studies, and human intervention trials, suggest that tea compounds, such as L-theanine, catechins, and tea pigments, flavonoids, may help prevent or treat depression. These effects are mediated through mechanisms such as inhibiting the activation of the HPA, regulating inflammation, promoting neurogenesis and neuroplasticity, modulating the weakened monoamine system, and improving gut microbiota disturbances (Figure 4) [79,80,81]. Specifically, regular consumption of green tea [82], jasmine flower tea [83,84], and ripened pu-erh tea [85], has been shown to reduce the risk of depression, likely due to their ability to restore intestinal homeostasis [86].

It has been observed that individuals who consume more tea have a lower risk of depression, particularly those who drink three cups daily, with a 37% reduced risk associated with this consumption [87,88]. It underscores the important role of various neuro-related tissues in the connection between tea consumption and mental disorders [89]. Clinical studies have shown that both Americans and Japanese who consume more than two cups of green tea per day experience a significant reduction in the incidence of Parkinson’s disease, which also benefits cognitive memory function [90]. Drinking green tea can ameliorate the rate of depression among the elderly [91]. Green tea consumption has been found to have a considerable reduction in depression, especially among elderly individuals, with severe depression dropping from 23.6% to 11.1%, and moderate depression reducing from 45.8% to 26.4% [92]. Moreover, studies indicate that depressed individuals administrated with green tea showed significant reductions in the scores on the Mullen Scales of Early Learning Nonverbal Developmental Quotient (MANDS) and the Hamilton Rating Scale for Depression-17 (HRSD-17) compared to those treated with a placebo [93]. Long-term green tea consumption has also been associated with reduced inflammation and increased estradiol levels in postmenopausal women, thereby lowering the risk of depression [94].

### 5.1. The Application of Tea and Tea Polyphenols in Alleviating Depression via Different Pathways

Study had proved that Ziyan Green Tea administration could stimulate the levels of 5-HT, BDNF, and DA in the brain, and reduce the inflammatory factors, IL-6 and TNF-α, to prevent depression induced by CUMS [95]. Drinking up to four cups of black tea and caffeine intake between 450 and 600 mg daily has also demonstrated protective effects against depression [96]. Jianghua Kucha black tea could increase the content of monoamine neurotransmitters (5-HT and DA), BDNF, decrease the inflammatory cytokines, like IL-6 and TNF-α in the brain tissue of CUMS mice [97]. Furthermore, oral administration of Matcha tea powder has been shown to activate the dopaminergic system, particularly the PFC-nucleus accumbens (NAc)-ventral tegmental area (VTA) circuit, exerting an antidepressant-like effect [98]. The treatment of jasmine tea can significantly improve CUMS-induced depressive behavior, and increase the concentration of neurotransmitters (BDNF, 5-HT), and reduce the levels of pro-inflammatory factors (TNF-α and NF-κB) [84].

The bioactive compounds in tea also exhibit potential in regulating depression, through various mechanisms, including modulation of signaling pathways, gut microbiota, inhibition of abnormal protein aggregation, normalization of hyperactivity the HPA, and antioxidant and anti-inflammatory effects [81,87,99]. Green tea polyphenols, known for their antioxidant properties, can penetrate the BBB after oral intake and play an important role in neuroprotection [100]. These polyphenols also exert antidepressant effects by regulating monoamine neurotransmitters and the HPA [93]. In socially defeated depression models, intervention with green tea polyphenols improved behavior via the hippocampal CREB-BDNF pathway [101], promoted the morphology of hippocampal neurons, reduced cell apoptosis, and increased levels of postsynaptic density protein 95 (PSD-95), synaptophysin (SYN), and phosphorylated (p)-CREB. Additionally, tea polyphenols regulate microglia polarization (M1/M2) and enhance BDNF expression in the hippocampus, thereby alleviating depression-like symptoms in rats with type 2 diabetes [102]. Green tea extract also has been safely administered to individuals with human immunodeficiency virus (HIV) on antiretroviral therapy, showing rapid improvement in depressive symptoms compared to a placebo [103]. Therefore, tea polyphenols possess anti-depressant function, and regular tea drinking may help reduce both the rate and severity of depression [104]. Studies have indicated that tea polyphenol interventions significantly alleviate depression symptoms by increasing levels of DA and 5-HT in the PFC and hippocampus of CUMS mice [56,105].

### 5.2. The Application of EGCG in Alleviating Depression via Different Pathways

**Figure 5:**
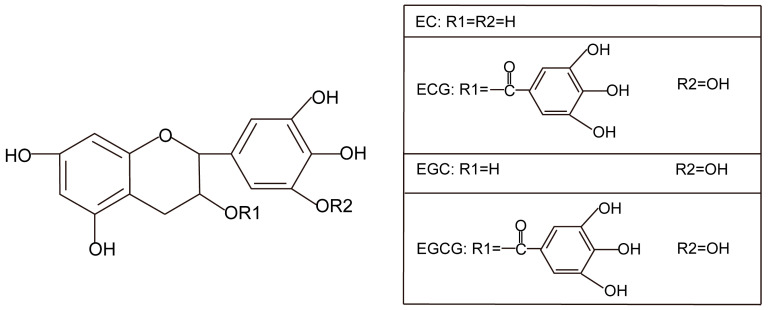
Structural formula of the four catechin components in tea polyphenols.

There are four important polyphenols in green tea, which are classified as catechins, including epicatechin (EC), epicatechin (EGC), epicatechin-3-gallate (ECG), and epicatechin-3-gallate (EGCG) (Figure 5). EGCG, which is the main catechins of tea polyphenol and reach to 10–15% [106], has a variety of physiological activities, such as anti-oxidation [107], anti-tumor [108], anti-aging [109], anti-depression [110] and so on.

EGCG, a natural polyphenolic antioxidant abundant in green tea, has shown considerable potential in alleviating depression [111]. EGCG can improve depression with a higher dosage of 3 g/d [112]. As a dietary supplement, EGCG significantly improves depressive symptoms [113] and, when combined with coconut oil, increases blood albumin concentration, reducing depression in patients with multiple sclerosis [114]. EGCG helps regulate depression and anxiety symptoms induced by high cortisol levels due to stress [115]. It also demonstrates neuroprotective effects against diseases such as Alzheimer’s and Parkinson’s [116,117]. In rat models of hippocampal radiation injury, EGCG exhibited neuroprotective effects by reducing apoptosis [118]. EGCG may restore HPA activity by upregulating ERK, decreasing levels of CORT, CRH, and ACTH, reducing neuronal overexcitation, and enhancing GABA transmission through activation of the SIRT1/PGC-1α pathway [80]. Administration of EGCG alleviated depressive-like behavior induced by CUMS, decreased 5-HT levels in the colon, and increased in the hippocampus, suggesting its potential for anti-depression by regulating 5-HT levels, enhancing intestinal barrier function, and providing neuroprotection in the hippocampus [119]. EGCG also promotes neuronal development and synaptogenesis in the CNS by activating CREB [120], a transcription factor that targets BDNF [121]. Furthermore, the administration of EGCG significantly improved the freezing response in contextual fear conditioning, alleviated cAMP response element-binding protein and BDNF related to memory in the hippocampus, reversed the change in allopregnanolone and progesterone levels in the brain, and mitigated the dysfunction of HPA [122]. In CUMS mice models, EGCG improved depression behaviors by regulating the BDNF/TrkB signaling pathway, reducing apoptosis, and protecting hippocampal neurons [123]. EGCG also exhibited anti-anxiety effects by regulating the HPA [124] and protected mice from nerve damage caused by CUMS while enhancing learning and memory functions [125]. EGCG administration in CUMS mice reduced serum CORT and ACTH levels, inhibited hippocampal malondialdehyde production, promoted glutathione peroxidase (GSH-Px) and superoxide dismutase secretion, and downregulated IOD, IL-6, and IL-1β [126]. The inhibitory effect of EGCG on cerebral edema secondary to traumatic brain injury in rats via decreasing the vascular permeability, increasing SOD, reducing MDA, and the expression of AQP4 and GFAP [127]. The antidepressant effects of EGCG also upregulate hippocampal BDNF mRNA and reduce CA3 neuronopathy [127]. It was found that after eight consecutive weeks of CUMS modeling, EGCG (200 mg/kg b.w) was given for two weeks, which could significantly reduce the depressive-like behavior induced by CUMS [128]. The expression level of BDNF mRNA in the hippocampal CA3 region was increased, and the morphological characteristics of CA3 neurons in CUMS mice were reduced, including apoptosis, myelin loss or destruction, and synaptic degeneration [128]. The therapeutic effect of EGCG on CUMS-induced behavior changes is comparable to that of the tricyclic antidepressant clopropramine hydrochloride (Anafenil) [128].

EGCG also suppresses immune and inflammatory responses and inhibits apoptosis, further mitigating depression by restraining the mRNA in the hippocampus [110,127,129]. Nabila E. Abdelmeguid demonstrated that the antidepressant action of EGCG involved downregulating serum IL-1β [128]. Similarly, Jinpeng Wang et al. found that EGCG alleviated anxiety by inhibiting neuroinflammation in the rat hippocampus and reversing alterations in STAT3 and IL-6 in the brain, thus preventing apoptosis in the hippocampus [110,129]. EGCG can improve the depressive behavior of CUMS mice by decreasing the activation of NLRP3 inflammatory, inhibiting the mTOR signaling pathway, repairing autophagy levels, attenuating the expression of apoptotic markers, and reducing abnormal changes in lipid levels [130]. EGCG intervention also alleviated the concentrations of IL-6 in serum and hippocampus of myocardial infarction and regulated caspases 3, 8, and 9 mRNA expression, which had a closer relationship to cell apoptosis in the hippocampus [129]. EGCG reduced the expression of STAT3, which may be associated with IL-6 [129]. EGCG/AA NPs, EGCG was combined with PEGylated poly(lactic-co-glycolic) acid NPs (nanoparticle) with ascorbic acid (AA), had better influence than free EGCG in decreasing motor disturbances and depression-like behavior related to 3-nitropropionic acid toxicity. EGCG/AA NP treatment also ameliorated neuroinflammation and prohibited neuronal loss [131]. Compared with the control group, EGCG-loaded nanosuspensions (25 mg kg^−1^) and EGCG–piperine nanocomplexes (25 mg kg^−1^) can significantly alleviate the antidepressant-like behavior of mice by reducing the levels of brain MAO-A, plasma nitrite, brain malondialdehyde, and brain catalase, and increasing the levels of CORT in plasma and brain reduced glutathione in both unstressed and stressed mice [132]. It has been found that EGCG administration can ameliorate the symptoms of anxiety and depression in mice Via reducing Sema3A and increasing phosphorylation of GSK3β in the hippocampus and can be used to treat Postpartum depression (PPD) [133].

### 5.3. Tea and EGCG Alleviated Depression by Regulating Gut Microbes

There are potential connections between the consumption of tea and its impact on gut microbiota, suggesting a role in alleviating symptoms of depression. Jasmine tea has been found to attenuate depressive symptoms induced by CUMS via the gut–brain axis, which has been proven to cause a relative abundance of *Firmicutes*, *Patescibacteria*, *Spirochaetes*, *Bacteroidetes*, *Elusimicrobia*, and *Proteobacteria* [83]. Ziyan green tea under low and high doses had a positive preventive influence on depressive symptoms induced by CUMS through altering the composition of gut microbiota, including *Corynebacterium*, *Faecalibaculum*, *Enterorhabdus*, and *Desulfovibrio*, improving the gut microbiota environment and then promoting the production of intestinal metabolites, changing the intestinal purine metabolism, bile acid biosynthesis, and cysteine metabolism [95]. Jianghua Kucha black tea treatment significantly changed the composition of the gut microbiota to improve depression induced by CUMS, mainly by increasing the abundance of *Turicibacter*, *Faecalibaculum*, *Akkermansia*, and *Desulfovibriom*, and reducing the abundance of genera *norank_f__Muribaculaceae* and *Lactobacillus* [97]. Meanwhile, the intervention of Jianghua Kucha black tea regulated the metabolites in fecal, mainly characterized by enriching the concentration of some secondary bile acids (BAs) and inhibiting the concentration of some purine intermediate metabolites [97]. The levels of SCFAs were increased after tea consumption and then penetrated the blood–brain barrier, which played a positive antidepressant effect in mice [134]. CUMS rats treated with Jasmine tea improve the ecological imbalance, improving the relative abundance of *Bacteroides*, *Cyanobacterium*, *Clostridium*, and *Lactobacillus*, inhibiting the abundance of *Ruminococcus* and *Butyrivibrio*. Meanwhile, the metabolites in the serum of CUMS rats were modified after Jasmine tea intervention, with 22-regulated and 18 down-regulated, which may be closely related to glycerophospholipid metabolism pathways, glycine serine and threonine metabolism pathways, and nicotinate and nicotinamide metabolism pathways [84].

Recent studies have indicated that there are potential connections between the consumption of EGCG and its impact on gut microbiota, suggesting a role in alleviating symptoms of depression. The gut microbiota plays a significant role in the pathophysiology of depression, exerting its effects through the GBA. The microbiota not only modulates GBA function but also enhances the absorption of polyphenols, which are crucial for maintaining intestinal barrier integrity and healthy gut microbiota. Moreover, gut microbiota metabolizes polyphenols into more bioactive forms that are readily absorbed, potentially contributing to the alleviation of depressive symptoms via the microbial GBA [55]. It has been found that EGCG can be metabolized by gut microbiota bacteria, e.g., to gallic acid, epigallocatechin, and (-)-epicatechin through ester hydrolysis [135]. The (-)-epicatechin formed epicatechin is then further converted to hydroxyhydroquinone. However, it is still unclear how these metabolites affected depression.

While polyphenols are absorbed directly in the small intestine, their complex structures often result in low bioavailability, with most reaching the large intestine unchanged. These polyphenols play a crucial role in modulating gut microbiota, influencing various metabolic processes in the host. EGCG, a major tea polyphenol found in tea, has antioxidant, anti-inflammatory, and neuroprotective properties that can influence the composition and function of the gut microbiota. By increasing the growth of beneficial bacteria and decreasing harmful bacteria, EGCG helps maintain a healthy gut microbiota balance. Research has shown that EGCG administration significantly increases diversity indices such as Chao1, phylogenetic diversity, Shannon, and Simpson in rats subjected to CUMS. Additionally, EGCG was found to increase the abundance of *Bacteroidetes* while reducing *Firmicutes*, leading to a decreased F/B ratio, indicating a shift in the composition of gut microbiota [136]. EGCG treatment was shown to alter the diversity and abundance of intestinal microorganisms in the stool of CUMS rats, further highlighting its therapeutic potential in modulating gut microbiota and its associated physiological impacts.

## 6. Conclusions

Depression poses a significant global health challenge characterized by its high risk, incidence, persistence, and tendency of recurrence, significantly impacting individuals’ quality of life worldwide. Recent findings have underscored the pivotal role of gut microbiota in modulating depression-like behaviors, influencing the HPA, monoamine neurotransmitters, immune-inflammatory responses, cellular signaling pathways, and metabolic processes.

The ability of gut microbiota to modulate levels of monoamine neurotransmitters has sparked interest in exploring the therapeutic potential of tea and its bioactive component, EGCG, for neuropsychiatric disorders. While individual tea compounds may not exert strong pharmacological effects on their own, their combined action, particularly of L-theanine and tea polyphenols, can synergistically impact various neurobiological systems, potentially reducing the risk of depression.

Further research is needed to better understand the synergistic effects and mechanisms of these bioactive substances, aiming to develop functional foods for the prevention and management of depression. Additionally, innovations such as nanotechnology may improve the bioavailability of EGCG, while fermenting tea with beneficial gut microbiota could further optimize the bioavailability of its compounds. Advanced research techniques, such as fecal microbiota transplantation, metabolomics, proteomics, and genomics should be employed to comprehensively assess how tea compounds affect depression.

Clinical studies, particularly in neurodegenerative diseases like Parkinson’s disease, are currently limited. More extensive clinical trials are needed to establish the efficacy and safety of tea and EGCG in treating neurodegenerative and neuropsychiatric disorders in humans. Comprehensive investigations into the safety profile of tea and its health benefits are essential for future research.

## Abbreviations

HPA, hypothalamic–pituitary–adrenal axis; EGCG, Epigallocatechin gallate; MDD, major depressive disorder; 5-HT, serotonin; NE, norepinephrine; GBA, gut–brain axis; CUMS, chronic unpredictable mild stress; CNS, central nervous system; ECCs, enteroendocrine cells; PSD, post-stroke depression; OTUs, Operational Taxonomic Units; SCFAs, short-chain fatty acids; SNS, sympathetic nervous system; ACTH, adrenocorticotropic hormone; CRH, corticotropin-releasing hormone; CORT, corticosterone; IBS, irritable bowel syndrome; DA, dopamine; GABA, gamma-aminobutyric acid; ENS, enteric nervous system; PYY, peptide YY; GLP-1, glucagon-like peptide-1; IDO, indoleamine-2,3-dioxygenase; PFC, prefrontal cortex; BDNF, brain-derived neurotrophic factor; ROS, reactive oxygen species; DAQ, 3,4-dihydroxyphenylacetaldehyde quinone; LPS, lipopolysaccharide; IL-1β, interleukin-1β; tumor necrosis factor-alpha (TNF-α), CREB, cAMP response element-binding; MAPK, mitogen-activated protein kinases; CAMK II, calcium/calmodulin-dependent protein kinase II, MANDS, Mullen Scales of Early Learning Nonverbal Developmental Quotient; HRSD-17, Hamilton Rating Scale for Depression-17; NAc, nucleus accumbens; VTA, ventral tegmental area; SYN, synaptophysin; PSD-95, postsynaptic density protein 95; HIV, human immunodeficiency virus; SOD, superoxide dismutase; GSH-Px, glutathione peroxidase, EGCG/AA NPs, EGCG was combined with PEGylated poly(lactic-co-glycolic) acid NPs (nanoparticle) with ascorbic acid (AA), PPD, Postpartum depression, BAs, bile acids.
